# NPC1 in human white adipose tissue and obesity

**DOI:** 10.1186/1472-6823-13-5

**Published:** 2013-01-30

**Authors:** Clara Bambace, Ingrid Dahlman, Peter Arner, Agné Kulyté

**Affiliations:** 1 Department of Medicine Huddinge, Lipid Laboratory, Karolinska Institutet, 141 86, Stockholm, Sweden

## Abstract

**Background:**

Genetic studies have implicated the *NPC1* gene (Niemann Pick type C1) in susceptibility to obesity.

**Methods:**

To assess the potential function of NPC1 in obesity, we determined its expression in abdominal white adipose tissue (WAT) in relation to obesity. *NPC1* mRNA was measured by RT-qPCR in lean and obese individuals, paired samples of subcutaneous (sc) and omental (om) WAT, before and after weight loss, in isolated adipocytes and intact adipose pieces, and in primary adipocyte cultures during adipocyte differentiation. NPC1 protein was examined in isolated adipocytes.

**Results:**

*NPC1* mRNA was significantly increased in obese individuals in scWAT and omWAT and downregulated by weight loss. *NPC1* mRNA was enriched in isolated fat cells of WAT, in scWAT versus omWAT but not modified during adipocyte differentiation. NPC1 protein mirrored expression of mRNA in lean and obese individuals.

**Conclusions:**

NPC1 is highly expressed in human WAT adipocytes with increased levels in obese. These results suggest that NPC1 may play a role in adipocyte processes underlying obesity.

## Background

Obesity is characterized by an excess of white adipose tissue (WAT) and marked adipocyte dysfunction that increase the risk for insulin resistance, type 2 diabetes mellitus and cardiovascular disease. White adipose tissue is not only energy reserve; it is also an active organ that contributes to whole body energy homeostasis by several different mechanisms. The underlying causes of obesity are complex and involve factors including caloric over-supply, a sedentary life-style, and a genetic predisposition.

A recent genome-wide association study report has indicated that the Niemann-Pick C1 gene (*NPC1*) is associated with early-onset and morbid adult obesity [1]. The NPC1 protein regulates transport of lipoprotein-derived lipid (cholesterol and fatty acids) from late endosomes/lysosomes to other cellular compartments and is responsible for maintaining intracellular cholesterol homeostasis [2-4]. However, the human genome-wide association study did not address how the *NPC1* gene variant promotes weight gain. Interestingly, a recent report showed that *NPC1+/−* male mice, when fed a high fat diet, deposited more fat and were heavier than their wild-type siblings in the absence of hyperphagia. They also developed adipocyte hypertrophy [5,6]. This data suggest that NPC1 has a previously unknown role in maintaining energy and metabolic homeostasis.

Our interest in NPC1 was stimulated by these findings, which imply a role for NPC1 in obesity and potentially adipose metabolism. We have previously reported that *NPC1* is highly expressed in human WAT [7]. In this study we investigated the hypothesis that NPC1 is active within human WAT. We performed a more detailed profiling of NPC1 mRNA and protein levels in relation to obesity and mRNA in relation to regional adipose depots and cellular origin.

## Methods

### Cohorts and clinical investigation

Investigated cohorts are described in Table 1. Cohorts and clinical investigation were previously described in details [7]. Obesity was defined as a BMI (body mass index) ≥30 kg/m^2^, whereas non-obesity was defined as BMI <30 kg/m^2^. Some subjects were examined before and 2–3 years after weight loss following either bariatric surgery or behavioral modification when a weight stable non-obese state had been reached. Subjects were recruited by local advertisement for the purpose of studying genes regulating obesity and fat cell function. Informed consent was obtained from all study subjects. The project was conducted in accordance with the guidelines in The Declaration of Helsinki and approved by the Regional Ethics committee in Stockholm, Sweden.

**Table 1:** Clinical characteristics of investigated subjects

**Cohort**	**Obese/overweight**			**Non-obese**		
	**Female/Male**	**Age (years)**	**BMI (kg/m**^**2**^**)**	**Female/Male**	**Age (years)**	**BMI (kg/m**^**2**^**)**
1. Obese/non-obese (sc)	64/22	39 ± 8	36 ± 6	47/11	36 ± 11	23 ± 3
2. Obese/non-obese (om)	22/0	43 ± 9	44 ± 4	13/0	40 ± 13	24 ± 2
3. Before/after weight loss	10/3	39 ± 7	40 ± 6			
4. sc/om	18/0	41 +10	45 + 4			
5. Cells/tissue	6/0	50 ± 13	36 ± 6	7/0	33 ± 10	23 ± 2
6. Differentiation	12/0	40 ± 8	27 ± 3			
7. Cells (proteins)	8/4	45 ± 8	36,6 ± 6,6	11/1	38 ± 11	23,2 ± 1,2

Values are presented as range (mean ±SD).

All subjects were healthy except for obesity and were investigated in the morning after a night’s fast. For those patients for which scWAT and omWAT were available, peroperative biopsies were obtained at the beginning of surgery. Only saline was given as intravenous infusion until adipose tissue was removed. For all other subjects, an abdominal subcutaneous WAT needle biopsy was obtained as previously described [8]. Tissue pieces were rapidly rinsed in saline and subsequently frozen in liquid nitrogen and kept at −70°C.

### Adipocyte experiments

Isolated fat cells were prepared according to the collagenase procedure as described [9]. Mean fat cell weight and volume were determined; lipolysis and lipogenesis experiments were conducted as described [10,11]. In some experiments we save the stroma fraction to prepare primary adipocyte cultures as previously described in details [12]. Briefly, WAT was washed, cut into small pieces and digested with collagenase for 1 h at 37°C. The collagenase-treated cell suspension was centrifuged at 200 x g for 10 min and the supernatant, containing mature adipocytes and collagenase solution, was removed. The remaining stroma vacular fraction (containing preadipocytes and mesenhymal stem cells) was resuspended in erythrocyte lysis buffer for 10 min, filtered though a nylon mesh and centrifuged as above. The supernatant was discarded and the pellet suspended in an inoculation medium DMEM/F12 supplemented with 10 % fetal bovine serum, 100 μg/ml penicillin-streptomycin and subsequently filtered through a 70 μm pore size filter. The cells were plated at the density of 30.000-50.000 cells/cm^2^ in inoculation medium to allow cells attachment. After 24 h the medium was changed to differentiation medium (DMEM/F12 supplemented with 15 mM HEPES, 100 μg/ml penicillin-streptomycin, 2.5 μg/ml amphotericin B, 66 nM human insulin, 1 nM triiodo-L-thyroine, 10 μg/ml human transferrin, 33 μM biotin, 17 μM panthotenate, 100 nM cortisol and 10 μM rosiglitazone (BRL49653). Rosiglitazone was included first 3–6 days and then removed from the differentiation medium. *NPC1* mRNA was quantified at day 4^th^, 8^th^, and 12^th^ of differentiation in primary adipocyte cultures.

### RNA isolation and quantification of gene expression

RNA extraction, measurements of concentration and purity as well as reverse transcription was carried out as described [7]. RT-qPCR was performed using the SYBR Green-based technology. *NPC1* was amplified with the primers 5’-AGCCAGTAATGTCACCGAAAC and 5’-CCGAGGTTGAAGATAGTGTCG or Taqman probes (Applied Biosystems, Foster City, CA). 18S amplified with described primers [13]. Relative *NPC1* expression calculated using the comparative Ct-method, i.e. 2^ΔCt-target gene^/2^ΔCt-reference gene^ with 18S as internal control. Levels of 18S did not differ between groups.

### Protein measurements

Proteins from isolated adipocytes were prepared as described [14]. Thirty five μg of total protein was separated by 7.5 % SDS-PAGE and Western blot was performed according to standard procedures. Primary antibodies against NPC1 were obtained from Life Span BioSciences, Inc. (Seattle, WA). Secondary antibodies were from Sigma-Aldrich. NPC1 protein was normalized to the most abundant protein band (bovine serum albumin) on the membrane visualized with Ponceau C (Sigma-Aldrich, St. Louis, MO). Images were analyzed using Quantity One Software (Bio-Rad Inc., Hercules, CA).

### Statistical analysis

Differences in adipose tissue expression of individual genes between two groups were analyzed by Student’s t-test. Correlations between *NPC1* expression and quantitative phenotypes were evaluated by regressions, when appropriate with BMI as additional independent parameter. Age was not correlated to *NPC1* expression.

## Results

### Adipose *NPC1* expression in obesity

**Figure 1:**
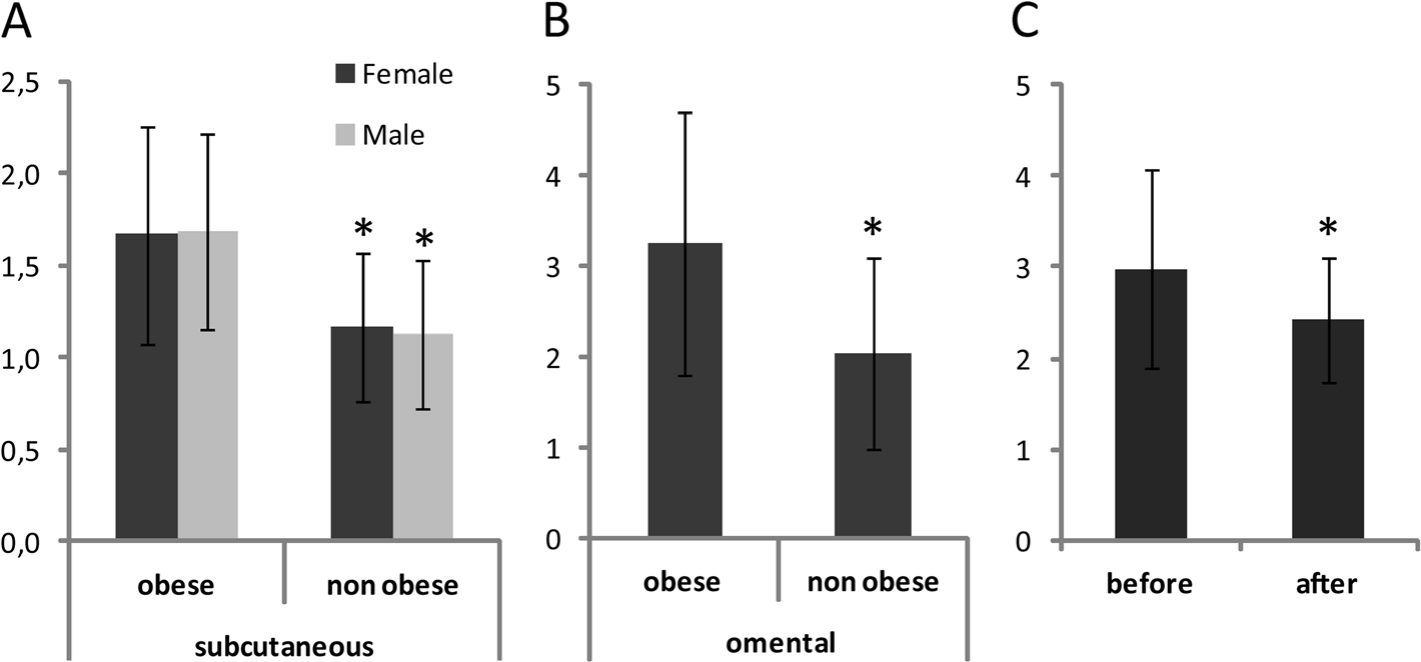
**Expression of *NPC1 *mRNA in subcutaneous and omental adipose tissue of obese vs. non-obese individuals and before/after weight loss. **Expression of *NPC1 *mRNA in (**A**) intact scWAT of obese (62 female and 22 male) and non-obese (47 female and 11 male) adults, (**B**) in intact omWAT of obese females (n=22) and non-obese females (n=13), (**C**) in adult females (n=10) and males (n=3) before and 2–4 years after intense anti-obesity treatment. Values are presented as range (mean ±SD), *P<0.05.

Both obese females and males displayed higher *NPC1* mRNA levels in intact pieces of abdominal scWAT (*P*=<0.0001 and 0.002, respectively), when compared to non-obese subjects (Cohort 1, Figure 1A). *NPC1* mRNA expression was also increased in omWAT (*P=*0.013, Cohort 2, Figure 1B) of obese females. We did not have access to male omWAT. *NPC1* mRNA levels were normalized by weight loss following either bariatric surgery or behavioral modification (*P=*0.0452, Cohort 3, Figure 1C). Age did not influence *NPC1* gene expression (values not shown).

**Figure 2:**
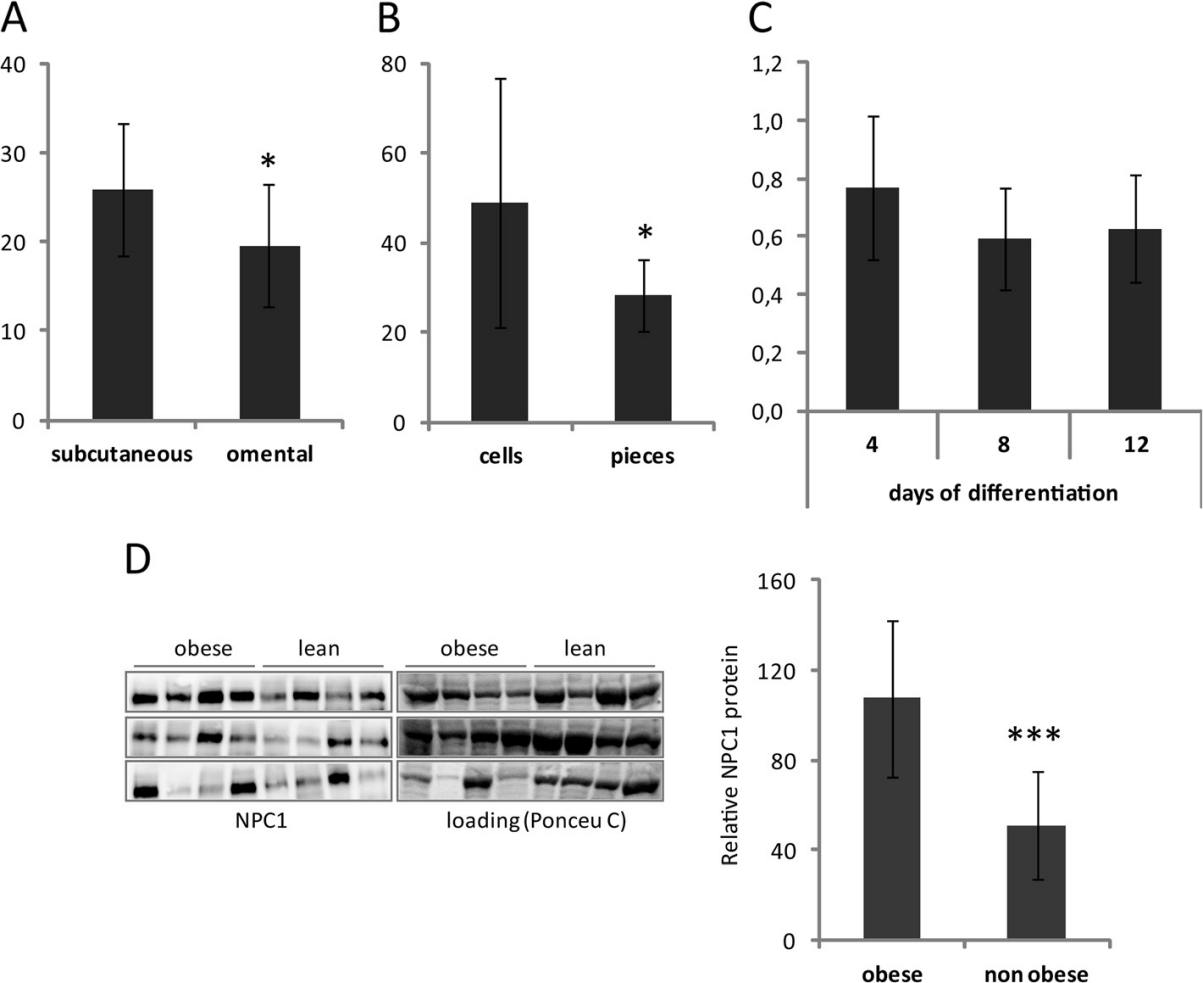
**Expression of NPC1 mRNA and protein levels in different fat depots and during differentiation. **Expression of NPC1 in **(A) **mRNA in intact scWAT and omWAT of obese females (n=18), **(B) **mRNA in isolated adipocytes from obese and non-obese adults (n=13), **(C) **mRNA at day 4, 8, and 12 of differentiation in primary adipocyte cultures from female (n=12), **(D) **NPC1 protein in isolated adipocytes from obese (n=12) and non-obese individuals (n=12). Each panel shows four obese and four non-obese individuals. Values are presented as range (mean ±SD), *P<0.05, ***P<0.001.

The adipose source of *NPC1* was also studied in more detail. *NPC1* mRNA was higher expressed in scWAT than in omWAT (*P=*0.0017, Cohort 2, Figure 2A). Moreover, *NPC1* mRNA levels were about two-fold higher in isolated fat cells as compared to intact adipose tissue pieces (Cohort 5, Figure 2B, *P*=0.018). However, *NPC1* expression was not altered during adipocyte differentiation as evidenced by the mRNA levels in primary adipocyte cultures at day 8 and 12 of differentiation relative to day 4 (Cohort 6, Figure 2C). To investigate if NPC1 protein levels reflect mRNA expression, we assessed NPC1 expression by Western blot in isolated fat cells of obese and lean subjects. Level of NPC1 protein was significantly higher in obese (*P*=0.0002, Cohort 7, Figure 2D).

**Figure 3:**
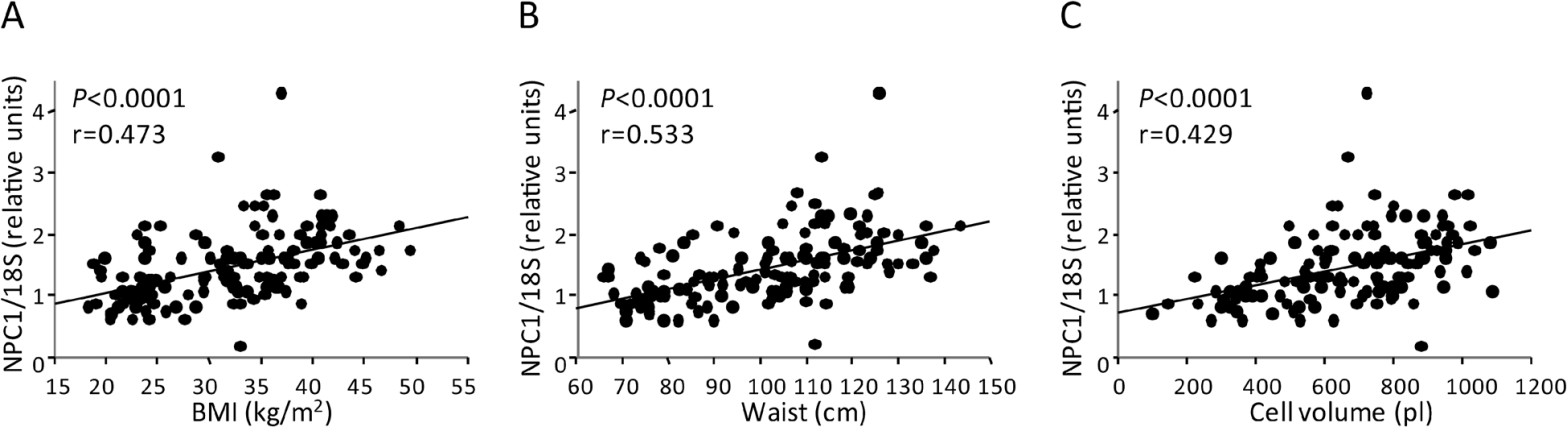
***NPC1 *expression in relation to BMI, waist circumference and adipose tissue morphology. **(**A**) BMI was correlated to *NPC1 *mRNA expression in scWAT (cohort 1), (**B**) waist circumference was correlated to *NPC1* mRNA expression in scWAT (cohort 1), (**C**) adipocyte volume was correlated to *NPC1 *mRNA expression in scWAT (cohort 1). *P *and r values are given for each simple regression plot. mRNA levels were normalized to the reference gene *18S *and are shown as relative units.

To further explore the role of NPC1 in WAT, the associations between scWAT *NPC1* mRNA levels and anthropological measurements, as well as phenotypes reflecting fat cell metabolism was determined (Figure 3). *NPC1* mRNA was positively correlated with BMI (*P*<0.0001, r=0.473, Figure 3A), waist circumference (*P*<0.0001, r=0.533, Figure 3B) and fat cell volume (*P*<0.0001, r=0.429, Figure 3C). There was no association between *NPC1* mRNA and levels of catecholamine-stimulated lipolysis or insulin-stimulated lipogenesis in adipocytes (data not shown).

## Discussion

The present detailed study of the obesity-gene *NPC1* revealed that *NPC1* mRNA levels were increased in both subcutaneous and omental fat depots, enriched in subcutaneous fat and isolated fat cells and down-regulated by weight loss. Adipose NPC1 protein mirrored the mRNA levels. Expression of *NPC1* was stable during *in vitro* adipocyte differentiation. There was no gender-specific difference on *NPC1* expression.

The correlation between *NPC1* mRNA levels with BMI and waist circumference in humans clearly fits into the concept of *NPC1* as an obesity gene. In agreement with this, humans affected by Niemann-Pick syndrome, with mutated *NPC1* gene, show impaired gain and maintenance of body weight [15]. However, in Niemann-Pick syndrome we do not know whether the *NPC1* gene has a specific influence on fat accumulation, or whether the impaired weight gain is an unspecific effect due to chronic disease. As regards experimental models, both female and male mice with decreased *NPC1* gene dosage gain weight on a high-fat diet [16]. On the other hand, *NPC1* gene is increased in livers of obese wild-type mice fed a high-fat diet [17]. This upregulation of *NPC1* in liver of obese mice mimic the effect we observe in human adipose tissue and could indicate that upregulation of NPC1 in obese subjects may be part of a pathway to counteract obesity. A similar paradoxical effect is observed for e.g. leptin, which counteracts obesity but still is increased in obese subjects [18]. We demonstrate no gender impact to *NPC1* expression in humans which is in concordance with the mice model.

Until recently, studies of *NPC1* gene function have been mostly focused on the pathogenesis of the CNS disease even though that is a secondary neurological impairment of lipid accumulation. Our results of increased NPC1 levels in adipose tissue of obese subjects and the consequent normalization after weigh loss, as well as the association between *NPC1* mRNA and fat cell size add impact on NPC1 function on visceral effects and support the concept of a metabolic function of *NPC1* gene in adipose tissue.

## Conclusions

NPC1 is enriched in fat cells of human adipose tissue, is elevated in obesity and affected by anti-obesity therapy. NPC1 may partially influence susceptibility to obesity by altering adipocyte function although further studies are needed to decipher its contribution to obesity development.

## Competing interests

The authors declare that they have no competing interests.

## Authors’ contributions

ID and AK designed the study. CB, ID and AK acquired, analyzed, interpreted the data and drafted the manuscript. ID and PA collected patient material. PA revised the final version of the manuscript. All authors read and approved the final manuscript.

## Pre-publication history

The pre-publication history for this paper can be accessed here:

http://www.biomedcentral.com/1472-6823/13/5/prepub
